# Is a Minimal Federal European Constitution for the European Union Necessary? Some Preliminary Suggestions Using Public Choice Analysis

**DOI:** 10.1007/s41412-022-00125-8

**Published:** 2022-06-20

**Authors:** Friedrich Schneider

**Affiliations:** Johannes Kepler University Linz, Research Institute of Banking and Finance, Altenbergerstrasse 69, 4040 Linz, Austria

**Keywords:** Public choice, European federal constitution, Federalism, Direct democray, Subsidiarity principle, D72, D78, H7, H11

## Abstract

After overcoming the Coronavirus Pandemic with massive spending programs and the possibility of running a deficit at the EU level, a minimal Federal European Constitution is proposed, to provide a legal and democratic base for the European Union. Six basic elements of a future European federal constitution are developed: the European Commission should be turned into a European government and the European legislation should consist of a two-chamber system with full responsibility over all federal matters. Three further key elements are the subsidiarity principle, federalism and direct democracy, which would provide the possibility for European voters to participate actively in political decision making, to break political and interest group cartels, and to prevent unwanted shifting of responsibilities from EU member states to the European federal level.

## Introduction

After successfully implementing European Economic and Monetary Union and overcoming the Coronavirus Pandemic we are currently facing several severe problems:

1. The urgently needed process of reforming some EU institutions, for example the unanimity voting rule, which blockades almost all decisions.
2. The need to constrain the behavior of politicians, so that further widening of the possibility of running a deficit at the constitutional level is limited.
3. The development of new institutional arrangements could lead to a Minimal European Constitution.^1^ Without major reform the EU could face a situation where the advantages of the EU are smaller than the disadvantages, with the consequence of destroying the EU. In order to avoid such a major crisis the author proposes the idea that some (minimal) European federal union will be necessary.^2^

Hence, in this paper some elements of a federal European constitution, such as subsidiarity, federalism, and direct democratic institutions, are suggested and justified with the help of constitutional economics and public choice analysis. In part 2 five basic elements of a European constitution are introduced and in parts 3 to 5 an attempt is made to scientifically justify these propositions, such as the design of European legislation (part 3), the subsidiarity and federalism principle (part 4), and direct democratic institutions (part 5). Finally, in part 6 a summary and some conclusions are given.

## Five Basic Elements of a Minimal Federal European Constitution (FEC)

The successful completion of European Economic and Monetary Union provided the opportunity to achieve a number of efficiency gains, and it also provided the opportunity to stimulate the growth rates of EU economies. Furthermore, competition has been promoted between member states by having a single currency and by weakening state-owned monopolies (such as power plants and telecommunication systems). However, there is also the danger that these positive influences would be weakened if national regulations were replaced by EU regulations, and even more importantly if a ‘new federal’ government at the EU level were to be ‘created’ without operating in a carefully designed institutional framework. Such a situation is now occurring, as the EU Commission expects to run large deficits for the next decade. All these developments could result in the EU Commission taking over more and more responsibilities from EU member states, which most of the single member states but also the majority of European citizens do not want.^3^

There should be careful consideration when creating such a Federal European constitution in order to prevent or limit a growing share of European federal government expenditure and the influence of interest groups. Buchanan (1990; pages 1 and 10) has already drawn attention in the early 1990s to the fact that ‘…Europe is now presented with a historically unique opportunity … The (constitutional) contract must be such as to ensure mutual gains from trade … The only constitutional structure that is consistent with the historically constrained setting of the 1990s is that of a federal union …’. Page 10 also stresses that ‘…a central political authority must come into power with some sovereignty over citizens in all of the nation-states’. According to Buchanan, it seems advisable to support the introduction of a Federal European Constitution (FEC).

It has already been stressed in Schneider (1993, 1996, 2009) and others (e.g. Mueller, 2003, and Eusepi & Schneider, 2004) that democratic systems with market economies, if unchecked, show a strong tendency towards increasing state activity at the highest level and interest group influence.^4^ As a consequence, the motivation of individuals to work efficiently, to engage in risky productive activities, and to innovate, is dampened. While the removal of intra-European barriers to the movement of people, goods, capital, and services might weaken the influence of special interest groups and bureaucracies in EU member states, growth in expenditure at the federal European level has to be expected as soon as Europe-wide interest groups and parties have been fully established. A European constitution thus would have to contain provisions to counterbalance such tendencies.

A European Constitution is also needed so that a European identity (at least) for certain issues which should be handled at the European level, can slowly grow or be formed. It has begun to grow since the creation of the Euro but on average European citizens feel primarily as, e.g. French, Italians or Germans, and not Europeans. This is one of the major difficulties when making suggestions about a European Federal Constitution, because in a European Constitution a safeguard against too much power of the federal level is missing.^5^ It is also difficult to create or strengthen the European identity as long as European voters have little or no influence in either changing the government or participating in certain major decisions of the European Union like widening the European Union, or a change in the finances. Therefore, the following constitutional elements would support the idea of a slowly growing (and/or creating) European identity, for example by introducing direct democratic elements in such a constitution.^6^

The following elements could be an essential part of such a constitution^7^:

I. The European Commission should be turned into a European government with strictly limited tasks (for instance, those set up in element II), and with the Council of Prime Ministers and Presidents in a second chamber (European Council) where each country has the same weight of voting power. Simple majority approval of both chambers (the European Parliament and the European Council) would be necessary for any legislation passed. Obviously, the European Parliament and the second chamber should solely have full authority and responsibility for all European budgetary and federal items. If the two chambers could not agree on a legislative or budgetary item, the parliament could overrule the decision of the second chamber by a qualified (for instance, 2/3) majority.
II. The jurisdiction of the European federal government should consist of those issues which, according to public finance theory, are best suited at the highest European Union level, e.g. defense, foreign policy, foreign trade policy, the enforcement of free intra-community movement (of people, goods, services and capital), anti-cartel and anti-monopoly policy and environmental policy concerning community-wide environmental problems. All these policy issues should only be taken over by the new European government if there is consensus between member states that the highest federal unit should do it, and if a referendum over these issues is approved by simple majority of the European voters and by simple majority of member states.
III. For the federal European government, it should not be possible to run or accumulate deficits on its current budget over a legislative period. If a budget deficit still occurs at the end of a legislative period, either expenditures should be cut or revenues should be increased, so that the budget will be balanced again. Longer (than a legislative period) lasting public debt at the European federal level should only be allowed for financing infrastructure expenditure or in crisis situations such as the Coronavirus pandemic, when huge funds are necessary to build up or improve infrastructure (e.g. health, education).^8^
IV. The activities of the Community should be financed by one specifically labeled tax, such as a proportional (indirect) tax or taxes on environmental issues. Changes in the rate of this tax should be subject to a 2/3 majority of the European parliament and of the second chamber, and to the approval of a popular referendum.
V. The institution of a popular referendum should be introduced for major policy issues (such as a change in the European constitution, change of tax rate, etc.). Furthermore, a popular referendum should be held if a certain number of voters ask for it and if at least a certain percentage of all people entitled to vote participate.^9^ The issue over whether a referendum should be held would only be accepted if it were approved by simple majority of European voters and by simple majority of member states.

## The Design of the European Legislation

The first element in Sect. 2 proposes the formation of a European government with a two-chamber system, the first chamber being the European parliament and the second chamber being the Council of Prime Ministers and Presidents, and both chambers obviously deciding all federal items delegated to the European Union. Some general remarks as well as Public Choice arguments will be made to justify this proposition. A number of studies (see e.g. Blankart and Mueller, 2003a, b and Testa, 2019) have analyzed the separation of powers from the perspective of positive constitutional economics. In two review articles Posner (1987) and Testa (2019) argue that the separation of powers increases the transaction costs of governing. This would hold for welfare-enhancing as well as for redistributive or even exploitative measures. The concept of separation of powers can be classified into horizontal separation (legislature, executive, judiciary) and vertical separation (federalism). The structure of isolated powers can vary to a considerable extent. Some progress has been made in analyzing the effects of separation of powers and political accountability. For example, Persson et al. (1997) show in a formal principal-agent model that separation of powers improves the accountability of elected officials, and thereby the utility of voters, but only under appropriate checks and balances. Two central provisions are needed: (1) there can be a conflict of interest between the executive and the legislative, and (2) moreover it must be impossible to implement any policy unilaterally, i.e. without the consent of both bodies. The basis for these results lies in the modeling of real-world political constitutions as ‘incomplete contracts’: ‘Elected politicians are not offered an explicit incentive scheme associating well defined payoffs with actions in all states of the world. Political constitutions only specify who has the right to make decisions, and according to which procedures for which circumstances. This makes it hard to tie specific rewards or sanctions to the contents of those decisions’ (Persson et al. 1997, p.5). The application of these results to the European Union makes it necessary that institutions are created, which leave only limited leeway for European Politicians to perform selfish actions.

The various effects of unicameral and bicameral legislators were first analyzed from a public choice perspective by Buchanan and Tullock in their famous book *Calculus of Consent* in 1962. One of the major conclusions in their analytical framework is that the optimal decision rule is that which leads to a minimum of the sum of external and decision costs (interdependence costs). Buchanan and Tullock (1962, p.235) conclude that ‘in comparison with unicameral systems, bicameral systems have higher decision costs …On the other hand, if the basis of representation can be made significantly different in the two houses, the institutions of bicameral legislature may prove to be an effective means of securing a substantial reduction in expected external cost of collective action without incurring as much added decision making cost as a more inclusive rule would involve in a single house’. The larger the majority required to reach a certain decision, the lower the external costs connected with that decision, because the number of opponents to a decision is negatively correlated with the required majority. On the other hand, it will become increasingly difficult to reach a decision at all, because decision costs are positively correlated with a required majority.

In more recent studies Testa (2019) and Voigt (2011) and a somewhat older Levmore (1992) investigate the advantages and disadvantages of bicameral versus unicameral systems. All conclude that a bicameral system might be better suited than a corresponding qualified majority in a unicameral system, to reduce the power of the agenda setter (mostly the government). Bicameral systems are often interpreted as a ‘break’ against overly active legislatures. Summarizing the effects of bicameral systems, one could conclude that the legislative activities in a bicameral system are indeed lower than in a unicameral one and this should be reflected in lower government consumption of economic output and higher growth rates.^10^

Some papers in constitutional economics (see, for instance, Moser & Schneider, 1997) try to analyze the consequences of a change in procedure on the power of European government organs.^11^ Within the European Union, the strengthening of the European parliament can be attributed as a further safeguard in addition to the second chamber. A bicameral system is also demanded, since it reduces the capability of rent seeking, because it is much more difficult to get a majority in both chambers than in only one.^12^ This is especially important after the widening of the European Union, because the more member states the European Union has, the more likely rent seeking might occur. The draft reports of the European Constitutional Group (1993, 2003, 2004) and Schneider (2009) stress the importance of competition as a mechanism to best fulfill consumer preferences. Competition, however, is not only crucial for the working of an economy; it is also needed in political markets, and the concept of institutional competition has a long tradition (starting with Tiebout’s ‘voting with the feet’). A better implementation of the distribution of powers, turning the Commission into a European government, the Council of Prime Ministers into a second chamber and strengthening the European parliament (which means giving these two chambers full legislative power), can be seen as a first step in applying the democratic principle to the European Union. This would be one way to reduce the political inefficiencies which are normally discussed when investigating the democratic deficits of the European Union.

## The Subsidiarity and Federalism Principles as Safeguards Against Government Growth and Centralization Tendencies

### The Subsidiarity Principle

One key element of a European federal union is a fixed set of tasks for the European federal government, which would have to be carefully designed (compare Kantorowicz, 2019, and Voigt and Gutman, 2019). This basic proposition comes from the idea of using the subsidiarity principle, which is in substance a constitutional norm. Vanberg (1994), Ostrom (1999) and Bickenbach (2013) argue that this norm is meant to provide a criterion for what can be considered as desirable constitutional order, a criterion that concerns the allocation of political authority in a multilayer system of states/governments. To put it simply, the subsidiarity principle requires that in a multilevel policy the distribution of power should be in favor of lower-level governments, and hence smaller jurisdictions. In other words, it demands that political authority always be located at the lowest possible level that is as close as possible to the citizens, the ultimate sovereign. Again, the consistent use of this principle is a necessary prerequisite for the functioning of a widened European Union in a productive way for all member states.

In the Commission report on the adaptation of existing legislation to the subsidiarity principle (European Commission, 1999, p.545) one reads ‘… the aim of the subsidiarity principle is, to see to it that decisions are taken as close as possible to the citizen, a constant watch being kept to ensure that action taken at community level is justified in the light of the means available to national, regional or local authorities’. Of course, the phrase ‘as close as possible’ is in urgent need of interpretation if the subsidiarity principle is to have specific normative content. The constitutional norm to allocate political authority in favor of more local levels of governments is in itself not a very operational instruction for the design of constitutional frameworks, and the question of how the general principle is to translate into more specific constitutional provisions is by no means a trivial issue.

Judgements on the preferability of particular constitutional arrangements (for instance, using the subsidiarity principle in a strict way) over others always refer to somebody to whom these alternatives are claimed to be preferable. In other words, all such judgements are directed to some addressee to whose interests they appeal. In democratic systems the ultimate addressees of constitutional proposals are, of course, the citizens who constitute this union. If the subsidiarity principle is claimed to be a desirable constitutional norm for the European Union, this means that such claims must be supported by arguments that can convince its citizens that it would be in their interests, if efforts in constitutional construction are guided by this principle. More precisely, these citizens would have to be convinced that adopting this principle would be in their constitutional interest, i.e. the interests that would form their choice, if it were up to them to select the rules for the polity in which they live.

What kinds of arguments could one put forward in support of the subsidiarity principle as a constitutional norm? In other words, what kind of arguments could be made in favor of this principle, when designing a federal European constitution? One major argument for this principle is the central concern on part of the members of any democratic organization about the principal-agent problem; that is, the issue of how one can ensure that power delegated to agents can, on the one hand, be used to the benefit of the principals and, on the other, be prevented from being used against the principals’ interests. As far as democratic politics is concerned there is a long tradition of inquiry, in political economy as well as in other social sciences, into the advantages of decentralization in political organizations. The results of this inquiry are of direct relevance to the subsidiarity principle.

However, it is obvious that the subsidiarity principle alone neither constitutes a basis to regulate intergovernmental relations in an enlarged European Union, nor does it protect the collectivities at the grass roots (Feld & Kirchgässner, 1996, see also Kantorowicz, 2019).^13^ Moreover, the authors argue that the introduction of the subsidiarity principle in the Maastricht Treaty has in fact shifted the burden of proof at least somewhat more toward the centralists; the notion of subsidiarity nevertheless remains very general and open to many interpretations. Hence, the use of the subsidiarity principle does not solve the dynamic organizational problem concerning under which conditions competencies or ‘rights’ should be given to lower governmental units. From a Public Choice point of view, there is a need for constitutional rules, which might prevent the ‘misuse’ of instruments by politicians, bureaucrats, and interest groups. Therefore, the subsidiarity principle must be ‘filled with life’ and the theory of federalism may represent an operational means to regulate the horizontal and vertical relationship between governmental units in the light of a potential Leviathan. To express it more sharpely this means that that the Federal institutions should embody the subsidiarity principle by explicit power conferring rules that (a) reserve certain powers to lower levels and (b) allow for the transfer of some of sub levels to higher organizational levels (perhaps sometimes accompanied with sunset clauses that require the renewal of transfer in fixed intervals respectively strike down referenda that must be held on these transferred power every x legislative periods.)

### Fiscal Federalism in a European Constitution

Federalism is an important institution that serves to establish competition within the political arena. Costs rise for voters as taxpayers if certain groups are able to appropriate the benefits of a publicly supplied good, but do not have to pay the full costs of its provision. This group can be the politicians and/or the bureaucrats, who are self-interested rent seekers, or special interest groups, who try to attain their goals. Although it is not argued here that politicians and bureaucrats always maximize their own utility up to the extent of actively exploiting the citizens and taxpayers, politicians and bureaucrats will do it from time to time, if they have the opportunity. Thus, federal competition provides another ‘safeguard’ against political decision makers taking advantage of their discretionary power in furthering their particular political views and interests (compare Mueller, 2003, and Kantorowicz, 2019).

Federal competition and federal institutions might also provide a very crucial argument in a future European constitution. As has been discussed, the highest federal unit in the European Union should only be given those tasks that bring additional benefits (for instance, due to EU-wide spillovers) to voters/citizens, if they are fulfilled by the highest federal unit, such as foreign defense policy and environmental policy. The restriction of these tasks is necessary so that a more-or-less automatic centralization of tasks (especially in the area of redistribution) at the highest federal level will be avoided. All other tasks should be provided by EU member states (at what level within the EU member states is not discussed here).^14^ The operating principles of a lively federalism can be summarized as follows^15^: as already argued, the European federal government would be constitutionally restricted in its domain of action – and severely so. Within its assigned sphere, however, the central government should be strong; sufficiently so to allow it to enforce economic freedom or openness over the whole of the EU territory. The EU member states would be prevented by the federal European authority from placing restrictions on the free flow of persons, resources and goods across their borders.

In order to guarantee, beyond the already suggested constitutional arrangements, that the central power does not take over either fiscal or other items from EU member states, Buchanan (1995) suggests an exit/secession option in the following way: EU member states must be constitutionally empowered to secede from the federal European Union. Secession, or the threat thereof, represents the only means through which the ultimate powers of the European federal government might be held in balance. In the absence of the secession issue, the federal European government may, by overstepping its constitutionally assigned limits, extract surplus value from the citizenry almost at will, because there would exist no effective means of escape.^16^ With an operative secession threat on the part of the EU member states, the European federal government could be held roughly to its assigned constitutional limits, while the EU member states could be left to compete among themselves in their capacities to meet the demand of citizens for collectively provided goods and services.^17^

Considering the arguments about federalism and subsidiarity, which policies should now be allocated at the European federal level? As proposed in part 2 in reference to the jurisdiction of the federal government, the European federal government should be responsible for competition policy, defense, and environmental policy, all those areas where one can expect EU-wide spillovers, so that individual EU citizen profits from them. The rationale for EU-wide environmental policy is first of all given by the global nature of some of today’s environmental problems: for instance, the danger of global warming by increased CO2 pollution.

## The Tax Base of the European Government

As already argued by Kirchgässner (1994) and Schneider (1993, 1996), the activities of the European government should only be financed by proportional (indirect) taxes. The rationale behind this idea lies in different control possibilities (coming from the Public Choice literature), which exist on different governmental levels.

First, any government will act more in accordance with the preference of individuals/voters, the more the citizens are able to control it. At lower governmental levels, with smaller communities, citizens have better possibilities of forcing the government to act according to their preferences; hence, this is another argument to assign government activities to the lowest possible level, again using the subsidiarity principle in a very strict way.

Second, as Feld and Kirchgässner (1995) argue, this implies that tasks as well as financial means that are easier to control are more suited to a higher governmental level than those that are difficult to control. The proposed indirect tax could only be changed by changing a law, which means that any change in the government share would have to be decided finding a majority in the parliamentary process and also via referendum, as suggested here (compare here e.g. the groundbreaking work of Winer, 2019). This ensures a public discussion, and at least as long as the European government seeks re-election it will hesitate to increase this tax, and it might face difficulties in getting an approvement by the parliament and the voters. Such proportional taxes leave relatively little room for leviathan behavior of a European federal government, especially if an increase in the tax rate has to be subject to a two-thirds majority of both chambers in the European parliament and the approval of a popular referendum. Therefore, at the European federal level only the revenues from this indirect tax should be available.

## Institutions of Direct Democracy in a Future European Constitution

Besides the important issues of federalism and subsidiarity, institutions of direct democracy such as popular initiatives and (obligatory) referenda could also be a crucial factor in a future European constitution. They should be seen as a necessary supplement to institutions of representative democracy such as the proposed two-chamber system and the European government.^18^

There is a second crucial institutional feature when introducing institutions of direct democracy. Referenda do not simply consist of a choice between given alternatives but should also be seen as an important ‘political education’ process over time. According to Frey (1994) and Frey and Bohnet (1994a, 1994b a,b) three stages can be differentiated. The first is the pre-referendum stage, in which the possibility of undertaking a referendum encourages discussion both among citizens and between politicians and voters. Pre-referendum discussion produces a number of important effects. Preferences are articulated, enabling mutually beneficial bargaining and exchange. Moreover, the agenda of alternatives is to a great extent determined by the citizens, thus constituting the relevant decision space. The pre-referendum stage screens the alternatives to be voted upon, reduces the number of relevant alternatives (often to only two) and makes the preferences somewhat more homogenous, thereby lowering the chance that the preference aggregation paradox will occur.^19^ The second stage is the formal decision situation, in which it can be seen that voters clearly express their content or discontent with a proposed referendum and quite often give a government a clear task to do. At the third, post-referendum stage, on the one hand as just argued the government has a clear task to do, and on the other hand, quite often, initiators of a referendum force the government to change their policy only by threatening to bring an issue to a popular referendum. But in some cases, the government can also undertake unpopular measures (like tax increases), if they are supported in a popular referendum.^20^

Institutions of direct democracy also have other important means, such as their possible use by voters to break politicians’ cartels directed against them. As Frey and Bohnet (1994a, 1994b) proposed, rent-seeking theory argues that representatives have a common interest in forming a cartel to protect and possibly extend political rents.^21^ Referenda and initiatives can be means to break politicians’ coalitions against voters. Initiatives require a certain number of signatures and if the initiators obtain these signatures they can force the government to undertake a referendum on a given (mostly disputed) issue. They are a particularly important institution, because they take the agenda-setting monopoly away from politicians and enable outsiders to propose issues for democratic decision, including those that many elected officials might have preferred to exclude from the agenda. As has been demonstrated in public choice theory, the group determining which propositions are voted on, and in what order, has a considerable advantage, because it decides to a large extent the issues that will be discussed and which ones will be left out.^22^ Referenda, whether obligatory or optional, enable voters to state their preferences to politicians more effectively than in a representative democracy. In a representative system, deviating preferences with respect to specific issues can only be expressed by informal protests, which are difficult to organize and to make politically relevant.

If one summarizes these findings, one can draw two conclusions. Accumulating research on the properties of popular referenda has revealed two major aspects on which institutional economics has to focus. One is the importance of discussion in the pre-referendum state (Frey, 1994). This implies that the number of propositions and the frequency of ballots must be low enough that voters have an incentive and the opportunity to collect and digest the respective information in order to participate actively in the decision.

The second element is that direct democratic institutions enable voters to break politicians’ and parties’ coalitions directed against them. Direct participation serves to keep the ultimate agenda-setting power with the voters. Initiatives and referenda are effective means by which voters might regain some control over politicians.

Hence, the introduction of direct democratic institutions such as the referendum at the highest European federal level in the European constitution is an absolute necessity, especially if the European federal government wants to change the tax structure or wants to take over a new policy field.^23^ This could only be implemented if approved by the legislation of the two chambers and by a popular referendum and if approved by a majority of states. Hence, the introduction of direct democratic elements would be crucial for a future European constitution so that the European government would keep strictly to its given tasks.

The introduction of direct democratic elements in a future European Constitution is supported by various researchers, like Feld (2003), Feld and Kirchgaessner (2003), Blankart and Mueller (2003a, b), Matsusaka (2019), and Winer (2019). In particular, the introduction of direct democratic elements could be an excellent tool in order to create a European identity. If European voters have a ‘say’ (e.g. to decide about European Union matters) they will be better informed about European affairs, they will discuss them, they will learn about them, and after some time they will decide in a European way, rather than just whether the issues are good for Italy, Germany or France. Therefore, the element of direct democracy is a very important ‘tool’ to ‘create’ an European identity.

## Conclusions

In this paper an attempt has been made to provide some basic elements of a future European federal constitution in order to provide a necessary framework for a functioning European Union with 28 members. Five basic elements have been put forward, such as that the European Commission should be turned into a European government, the Council of Ministers into a second chamber, and that the European Parliament should get full control and responsibility over all federal items together with the chamber. The jurisdiction of the European federal government should consist of a few specific items which are best suited to the highest federal level, such as foreign policy, defense and environmental policy. The activities of the federal European government should be financed by one proportional (indirect) tax, and direct democratic institutions should be introduced in a European federal constitution, such as the possibility to force the European government to set up a referendum. These elements are then justified by arguments dealing with the subsidiarity principle, the idea of federalism, and the effects of direct democratic institutions. As has been demonstrated, these elements are best suited to limit the domain of central European authority in the long run, even in the face of the strong tendency to centralization in nearly all federal states that has been observed in recent decades.

The idea is also proposed that the constitution should be structured in such a way that any attempt at future concentration of government activities at the European federal level would most likely be prevented by explicitly assigning specific governmental functions to each level of government and putting in additional safeguards, like the subsidiarity principle and direct democratic institutions, so that the federal European government could not take over tasks which were not approved by the majority of voters and European member states. From another perspective it would also be very difficult for the European government to take over additional tasks, because the necessary widening of the tax base could only be done if a majority of European voters/taxpayers were to approve it and also a majority of member states. Neither could the European government accumulate large deficits, which might provide another safeguard to limit the domain of a future central authority in Europe.

Two central features—direct democracy and fiscal federalism—should be key principles in a future European constitution. These have been shown in other federal units (like Switzerland and the United States) to be strong safeguards against policies that are not in line with a majority of voters’ preferences. Moreover, direct democratic elements provide the possibility for European citizens in a federal state to participate actively in political decision making, to break politicians’ and interest groups’ cartels, and prevent a shifting of responsibilities from EU member state level to EU federal government level. Proper assignment of tax competencies may also help to restrain centralization. If, as is suggested here, the tax competencies were to lie within the EU member states—with the exception of one specific indirect tax for the European government—such an element should also work.

With respect to the actual crisis facing the European Union in 2021, only a simple and rudimentary constitution could help to overcome the fears of the majority of European citizens, that the EU is not functioning well and could break altogether. Since, according to Downs (1957), voters have rational ignorance, it is necessary to build the European Union such that beside the advantages of monetary union, some additional advantages can be perceived by ordinary citizens, even if they are not well informed. Up to now, however, the advantages of the European Union have been very indirect and often not at all obvious to citizens, while public discussion has focused on the interests of producer interest groups and the influence of the Brussels bureaucracy. Thus, today the political opinion of ordinary citizens about the European Union varies between apathy and refusal. If such a simple and constitutional perspective were to be provided, and understood and accepted by the majority of European citizens, then actual political crises could be overcome. However, this would need quite drastic political change. European member state governments and EU political actors need to take much more serious action on the European issues (environmental issues, (illegal) immigration, etc.), which are of great concern to European voters, and would have to convince their voters that they could solve these problems with the help of a minimal European Constitution.

## Footnotes

1 The idea of a European Constitution is also discussed and mostly supported by various other researchers such as Buchanan (1990), Piris (2000), Feld (2003, 2005), Feld and Kirchgaessner (2003), Blankart and Mueller (2003a, b), and Vaubel (2004). Newer literature on this topic can be found in edited volumes by Eusepi and Wagner (2018), and Congleton, Grofman, and Voigt (2019), especially in Volume 2, part V ‘Constitutional Political Economy’.

2 Such a much less ambitious, smaller constitution, which takes the preferences of the European voters into consideration, has been developed by the European constitutional group; compare Bernholz, Schneider, Vaubel and Vibert (2004). Together with Giuseppe Eusepi the author already researched this topic in 2004, compare Eusepi and Schneider (2004). The author of this paper supports the idea that the EU should be transformed from a confederation to a minimal federation. Consequently, this paper is a revised version of Schneider (2002, 2009), where the idea of a European Federal Constitution was developed.

3 Compare Alesina and Perolti (1994), Alesina and Rodrick (1992),Kirchgaessner and Pommerehne (1995), Vaubel (1996), Voigt (2003), Feld (2005), consider also Eusepi and Wagner (2017, 2018), Congleton, Grofman, and Voigt (2019).

4 Compare the studies by Olson (1982, 1983), Bernholz (1990a, 1990b a,b, 1993), Schneider and Frey (1988), Peden and Bradley (1989), Weede (1986, 1990) De Haan and Siermann (1995, 1996), Holcombe (2019), Kantorowicz (2019), and Winer (2019).

5 Compare e.g. Feld (2003, 2005) and Abromeit (1998), and Schneider (2009).

6 Compare e.g. Feld (2003, 2005), Feld and Kirchgaessner (2003), Blankart and Mueller (2003a, b), and Matsusaka (2009).

7 The justification of these six (?) – the numbering goes only to “V” below– elements is given in the following points and for a more intense discussion of constitutional issues, see Gwartney and Wagner (1988), Vanberg and Buchanan (1989), Kirchgässner (1994), Oates (1985), Blöchliger and R.L. Frey (1992), Eichenberger (1994), Feld (2005), Vaubel (2004), and Congleton, Grofman and Voigt (2019).

8 Whether the European economic and monetary integration provides incentives to increase public deficits is investigated in Horstmann and Schneider (1994). Compare also Feld and Kirchgaessner (1997), see also Wagner (2015), DelliSanti and Wagner (2017), and Brennan (2018, 2012).

9 The precise conditions under which a referendum would be held and accepted would have to be specified; the main point is that the option would exist to force the European government to hold a referendum over a certain issue.

10 A similar conclusion is reached, and preliminary empirical evidence is given, by the studies of Feld and Kirchgässner (1996) and Feld and Savioz (1997); for a more general view of this aspect see Weingast (1995) and Grofman, Brunell and Feld (2012).

11 Compare Peters (1996a, 1996b a,b), Steunenberg (1994), Hosli (2010), Congleton, Grofman and Voigt (2019), and Plechanovova et al. (2019).

12 The introduction of a bicameral system not only reduces rent seeking attempts but is also an important element in strengthening the federal structure of the European Union. The second chamber, in which the Prime Ministers and Presidents of every EU member state have equal voting power, can be seen as an institution which solely represents the interests of the EU member countries, like the German Bundesrat or the Schweizer Ständerat. It might especially help to take care of the interests of the large number of smaller EU member countries after the enlargement.

13 In this context it is not surprising that Delors (1992, p.12) argues that ‘subsidiarity does not enact any restriction for the Commission to take political action’ and he continues to argue ‘solely on the basis of the Maastricht Treaty subsidiarity is not judiciable’. See also Vaubel (1993, 1995, 1996) and Möschl (1993, 1995).

14 One could go a step further and put forward the idea of federal competition between and within EU member states when providing goods and services, but also financing them. Compare here the unorthodox idea of Bruno Frey (2019) about competition within the EU and the member states. Within countries it has been shown in extensive research on Switzerland by Kirchgässner and Pommerehne (1995), Pommerehne, Kirchgässner, and Feld (1995), and Feld et al. (2017) that there is extensive tax competition in Switzerland.

15 Compare here the groundbreaking studies by Brennan and Buchanan (1980), and Brennan (2012, 2018).

16 Compare here again the pioneering work of Buchanan and Faith (1987) and Buchanan (1991, 1995).

17 The (threat of) secession should be seen here as an ultimate ‘weapon’ for every EU member country in order to avoid a development that is not wanted by EU member states and their citizens. In principle it gives single countries power over (for them) critical issues and if it is used one might end up in a prisoner’s dilemma situation. But as the execution of the secession for a single EU country is not so easy (for instance, a qualified majority of voters in that country is necessary) it is unlikely that this instrument will be used frequently only for tactical reasons. In terms of game theory, the threat is unlikely to be credible.

18 Compare Feld (2003), Feld and Gebhard (2000, 2001a, 2001b), Eusepi (2004), Feld and Schaltegger (2005), Feld, Schaltegger and Schnellenbach (2008), and Matsusaka (2019).

19 Compare Frey and Bohnet (1994a, 1994b a,b) and Frey (1994).

20 For instance, a referendum might help the government to undertake unpopular measures in environment policy, compare Frey and Schneider (1997).

21 The literature on rent seeking was developed by Tullock (1967) and a survey is provided by Tollison (1982); see also Besley (2007), Dreher and Lang (2019).

22 Compare Denzau (1995) and Mueller (1987, 1989).

23 Such a conclusion is also reached by Bernholz (1990b), Feld and Kirchgaessner (1996) and Vaubel (1993, 1995).
